# Brimonidine is neuroprotective against glutamate-induced neurotoxicity, oxidative stress, and hypoxia in purified rat retinal ganglion cells

**Published:** 2010-02-17

**Authors:** Kelvin Yoon Chiang Lee, Mao Nakayama, Makoto Aihara, Yi-Ning Chen, Makoto Araie

**Affiliations:** 1 Department of Ophthalmology, University of Tokyo, Graduate School of Medicine, Tokyo, Japan; 2 Singapore National Eye Centre, Singapore

## Abstract

**Purpose:**

To investigate the neuroprotective effect of α2-adrenergic agonist brimonidine in the presence of glutamate-induced neurotoxicity, oxidative stress, and hypoxia on in vitro cultures of purified rat retinal ganglion cells (RGCs).

**Methods:**

Purified RGC cultures were obtained from retinas of 6–8-day old Wistar rats, following a two-step immunopanning procedure. After 72 h of cultivation, the neuroprotective effect of brimonidine (0.01 μM, 0.1 μM, and 1 μM) was investigated by culturing the RGCs under glutamate, oxidative, and hypoxic stress for a further 72 h, 24 h, and 12 h, respectively. Glutamate neurotoxicity was induced by adding glutamate (25 μM), while oxidative stress was induced by substituting the culture medium with B27 supplement without antioxidants, and hypoxia was induced by cultivation in a controlled-atmosphere incubator with oxygen levels 5% of the normal partial pressure. The RGC viability under each stress condition normalized to that under normal condition was evaluated as live cell percentage based on a total of 7–8 full repeated experiments.

**Results:**

The cell survival percentages of cultures exposed to glutamate, oxidative, and hypoxic stress were 58.2%, 59.3%, and 53.2%, respectively. Brimonidine dose dependently increased RGC survival in the presence of glutamate (80.6% at 1 µM), oxidative (79.8% at 1 µM), and hypoxic (72.3 and 77.4% at 0.1 and 1 µM, respectively) stress. In the presence of α2-adrenergic antagonist yohimbine (10 μM), brimonidine (1 μM) showed no protective effects on RGC viability.

**Conclusions:**

At a concentration of 0.1 µM or higher, brimonidine increased survival of purified rat RGCs in the presence of glutamate neurotoxicity, oxidative stress, and hypoxia. The neuroprotective effect of brimonidine is mediated via α2-adrenergic receptors at the RGC level.

## Introduction

Glaucoma is the second leading cause of blindness in the world, and various mechanisms of glaucomatous optic neuropathy (GON) have been thought to cause retinal ganglion cell (RGC) death leading to visual loss [1]. Elevated intraocular pressure (IOP), ischemia, elevated glutamate levels, excessive production of nitric oxide and free radical generation, oxidative stress and deprivation of neurotrophic factors can trigger the apoptotic mechanisms in RGCs, and a combination of these factors would lead to RGC apoptosis in glaucoma [2-8]. Hence, an ideal neuroprotective drug should be able to target the multiple apoptotic pathways triggered by these factors.

Brimonidine is a highly selective α2-adrenergic receptor agonist [9]. Brimonidine lowers IOP by reducing aqueous humor production and also by stimulating aqueous humor outflow through the uveoscleral pathway [10]; it is an IOP-lowering drug that is widely used to manage glaucoma patients [11-13]. Brimonidine has also been found to have a neuroprotective effect beyond IOP lowering. Animal models of optic nerve injury, ocular hypertension, and retinal ischemia have been used to demonstrate the neuroprotective effect of brimonidine [4,14-17]. However, in these in vivo studies where drugs were applied either topically or systemically, it was difficult to determine if the observed effects were attributable to direct effects on RGCs or indirect remote effects of the drug on inflammatory mediators, local blood supply, or other ocular tissues.

Because of the wide use and importance of brimonidine as an antiglaucoma drug and its potential in retarding the progression of glaucomatous visual field damage of open angle glaucoma patients through action beyond IOP reduction [18], further characterization of the neuroprotective effect of brimonidine has been assessed, particularly at the level of the RGC. In vitro studies with purified rat RGC cultures have been previously used to determine the neuroprotective effects of β-adrenergic antagonists and calcium channel blockers in various stresses, including hypoxic and oxidative stress [19-21]. Hypoxia has been reported to induce release of glutamate from isolated retina or cultured retinal cells as well as to activate the caspase cascade leading to RGC apoptosis [22-25]. Hypoxia-induced RGC death in the in vitro purified RGC model has been suggested to be mostly independent of excitotoxicity through glutamate receptors [19]. In vivo, however, glutamate levels may be increased from release by other neuronal and/or glial cells or dysfunction of glutamate uptake by glial cells [26]. The retina and its neurons consuming high oxygen and exposed to high levels of light are prone to oxidative stress, which leads to an increase in reactive oxygen species and possibly cell damage from influx of Ca^2+^ [2,27-30].

The aim of our study is to examine the neuroprotective effect of brimonidine against glutamate-induced neurotoxicity, oxidative stress, and hypoxia, using purified rat RGC cultures.

## Methods

### Materials

All animal studies were in compliance with the Association for Research in Vision and Ophthalmology (ARVO) Resolution on the Use of Animals in Research. Poly-L-lysine, BSA (BSA), L-glutamine, human recombinant brain-derived neurotrophic factor (BDNF), rat recombinant ciliary neurotrophic factor (CNTF), and yohimbine hydrochloride (Y-3125) were obtained from Sigma (St. Louis, MO). The papain dissociation system was from Worthington Biochemical (Lakewood, NJ); mouse antirat SIRP (CD172a) monoclonal antibody (MAB 1407P), and mouse antirat and mouse Thy1.1 monoclonal antibody (MAB 1406) were obtained from Chemicon International (Temecula, CA). The live/dead viability cytotoxicity kit (L-3224) was obtained from Molecular Probes (Eugene, OR). Brimonidine tartrate was obtained from Allergan, Inc. (Irvine, CA). B27 supplement minus antioxidants (AO-) was from Gibco (Grand Island, NY). Unless named, B27 supplement was with antioxidants.

### Purified rat retinal ganglion cell culture

RGC cultures were obtained from the retinas dissected from enucleated eyes of 6–8 day-old Wistar rats (Saitama Jikken Dobuts, Saitama, Japan), euthanized by inhalation with CO_2_, following the two-step immunopanning procedure as follows [31,32]. Tissue was incubated at 37 °C for 30 min in 15 U/ml papain solution and 70 U/ml collagenase in Hanks' balanced salt solution containing 0.2 mg/ml BSA and 0.2 mg/ml DL-cysteine. To yield a suspension of single cells, the tissue was then triturated sequentially through a narrow-bore Pasteur pipette in a solution containing 2 mg/ml ovomucoid, 0.004% DNase, and 1 mg/ml BSA. After centrifugation at 120× g for 5 min, the cells were rewashed in another ovomucoid-BSA solution (10 mg/ml of each). After centrifugation, the cells were resuspended in 0.1% BSA in phosphate-buffered saline (PBS, Sigma). Antibodies were removed, and the cell suspension was incubated in the anti-macrophage antibody-coated flask for 1 h. Cells adhering to the tube (RGCs) were resuspended in serum-free neurobasal medium (Gibco) supplemented with 2% B27 supplement, BDNF (40 ng/ml), CNTF (40 ng/ml), and forskolin (10 μM) and seeded onto 13 mm coverslips placed within 24 well plates. The coverslips had been autoclaved and coated with 0.05 mg/ml of poly-L-lysine (Sigma) overnight, rinsed twice with Hank’s buffered saline solution (HBSS), and then coated for 2 h with 1 μg/ml of laminin (Gibco). RGCs were cultured for 72 h under normoxic conditions (20% O_2_, 5% CO_2_, 75% N_2_ at 37 °C) before each experiment in serum-free B27 complete medium containing neurobasal medium (Gibco) with 1 mM L-glutamine (Sigma), B27 supplement (Gibco), 40 ng/ml BDNF, 40 ng/ml rat CNTF, and 10 μM forskolin. After completing 72 h of cultivation, RGCs were subjected to the following:

### Glutamate neurotoxicity

Control coverslips were moved to freshly prepared neurobasal medium containing B27 supplement and placed in normoxic conditions without glutamate. Test coverslips for glutamate neurotoxicity were then transferred to freshly prepared neurobasal medium containing both B27 supplement and glutamate (25 μM). These were then cultivated for a further 72 h.

### Oxidative stress

Control coverslips were moved to freshly prepared neurobasal medium with B27 supplement normally containing potent antioxidants (reduced glutathione, vitamin E, vitamin E acetate, catalase, and superoxide dismutase), while coverslips for oxidative treatment were transferred to neurobasal medium containing B27 without these five antioxidants (AO-), which induced oxidative stress [33,34]. The RGCs were further cultivated for 24 h.

### Hypoxic stress

Control coverslips were moved to freshly prepared neurobasal medium with B27 supplement and placed in normoxic conditions, while hypoxic stress was induced by placing the cultures in a hypoxic environment (controlled atmosphere of 5% O_2_, 5% CO_2_, 90% N_2_ at 37 °C) for 12 h.

### Application of brimonidine

Seven repeated full experiments were performed using three concentrations (0.01 μM, 0.1 μM, and 1 μM) of brimonidine; these were added separately to each of the test cultures.

### Effect of brimonidine in the presence of yohimbine

We studied the effect of yohimbine (10 μM), a specific α2-adrenergic receptor antagonist on the neuroprotective effect of brimonidine (1 μM) by adding brimonidine alone, brimonidine with yohimbine, and yohimbine alone to RGCs cultured under glutamate neurotoxicity, oxidative stress, and hypoxia. Eight separate, repeated, full experiments were performed with yohimbine.

### Assay of retinal ganglion cell survival rate

At the end of cultivation, the surviving RGCs were processed for viability by labeling with calcein-AM (2 μM), a component of the live/dead viability/cytotoxicity kit [32]. Live RGCs were defined as having a calcein-stained cell body with neurites extending at least 3 cell diameters from the cellular body. The RGC viability was calculated from two wells, those with exposure to the insults and the control group. The RGCs were counted manually in a total of eight fields of standardized location at 10× magnification. Live RGCs in each well were expressed as a cell survival percentage of the control culture with control medium. The average cell survival percentage of seven to eight experiments for each condition was expressed as the mean±standard deviation (SD).

### Statistical analysis

Dunnett’s test was used to determine if test groups were significantly different from controls. A p value of <0.05 was considered significant.

## Results

### Neuroprotection against glutamate neurotoxicity

**Figure 1:**
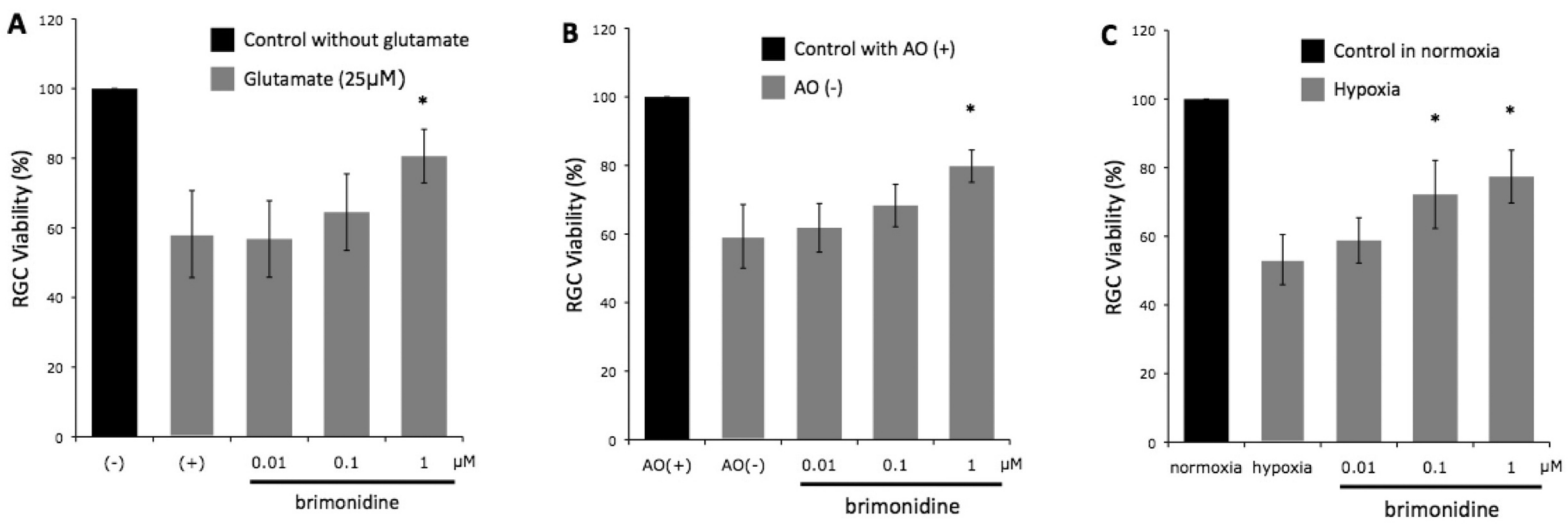
Retinal ganglion cell viability of seven experiments under (**A**) glutamate-induced neurotoxicity, (**B**) oxidative stress, and (**C**) hypoxic stress, with increasing concentrations of brimonidine. Brimonidine at a concentration of 1 μM significantly increased retinal ganglion cell (RGC) viability in all three stresses. Abbreviations: (-) represents control RGC cultures without glutamate; (+) represents control RGC cultures with glutamate, AO(+)represents medium with anti-oxidant; AO(-) represents medium without anti-oxidant, * represents p<0.01. n=7. Error bar indicates SD.

After completing 72 h of cultivation in the presence of glutamate alone, 58.2±12.5% of the RGCs survived compared to controls (Figure 1A). In the presence of 0.01 μM, 0.1 μM, and 1.0 μM of brimonidine, RGC survival was 56.8±11%, 64.5±11%, and 80.6±7.7%, respectively (n=7, p=0.990, 0.564, and 0.002, respectively).

**Figure 2:**
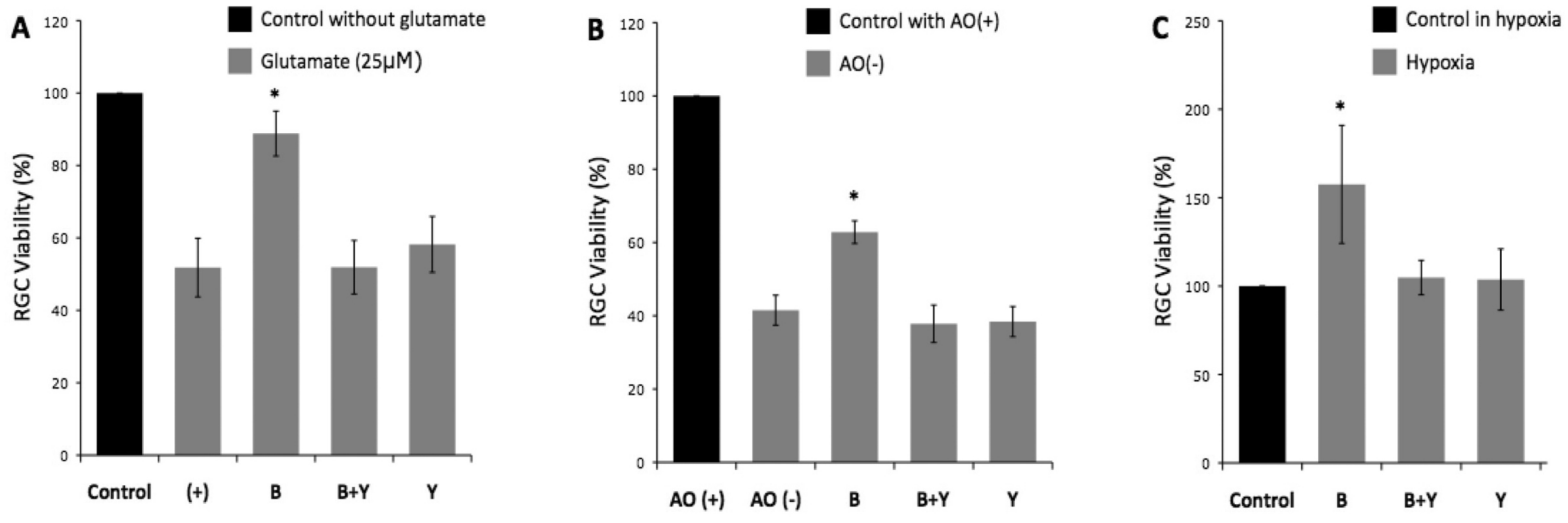
Retinal ganglion cell viability of eight experiments under (**A**) glutamate-induced neurotoxicity, (**B**) oxidative stress, and (**C**) hypoxic stress. The neuroprotective effect of 1 μM brimonidine was reproduced in all three stresses. The effect of brimonidine on the increase in retinal ganglion cell (RGC) viability was blocked by the α2-adrenergic antagonist yohimbine in all three stresses. Abbreviations: (+) represents control RGC cultures with glutamate; AO(+) represents medium with anti-oxidant; AO(-) represents medium without anti-oxidant; B represents RGC cultures with brimonidine added; B+Y represents RGC cultures with brimonidine and yohimbine added; Y represents RGC cultures with yohimbine added; *, p<0.001; n=7. Error bar indicates SD.

In the yohinbine experiments, the neuroprotective effect of brimonidine at 1.0 μM against glutamate neurotoxicity was replicated (p<0.001). RGC survival in the presence of brimonidine and yohimbine and of yohimbine alone was 51.9±7.4% and 58.2±7.7%, respectively (Figure 2A); these were not statistically different from controls with only glutamate (p=1.00 and 0.220).

### Neuroprotection against oxidative stress

In the presence of oxidative stress (AO-), RGC survival was reduced to 59.3±4.1% compared to the AO+ control group in normal cultivating conditions (Figure 1B). In the brimonidine AO- group, RGC survival was 61.8±7.1%, 68.3±6.2%, and 79.8±4.7% for brimonidine concentrations of 0.01 μM, 0.1 μM, and 1.0 μM, respectively (n=7, p=0.845, 0.064, and <0.001, respectively).

In the yohinbine experiments, the neuroprotective effect of 1.0 μM brimonidine against oxidative stress was replicated (p<0.001, Figure 2B). In the AO- group with brimonidine and yohimbine, 37.8±5.1% of RGCs survived, which was not significantly different from the AO- control group (p=0.215). Yohimbine alone did not significantly alter RGC survival (38.4±4.1%) compared to AO- controls in conditions of oxidative stress (p=0.342).

### Neuroprotection against hypoxia

Under hypoxic conditions (Figure 1C), RGC survival was reduced to 52.4±6.2% compared to the control group (Figure 1C). In the brimonidine group, RGC survival was 57.6±5.9%, 72.3±9.9%, and 77.4±7.7% for brimonidine concentrations of 0.01 μM, 0.1 μM, and 1.0 μM, respectively (n=7, p=0.762, 0.004, and <0.001, respectively).

In the yohinbine experiments, the neuroprotective effect of brimonidine against hypoxia was replicated (p<0.001, Figure 2C). In the presence of yohimbine, the RGC survival was not significantly increased by brimonidine (p=0.926). Yohimbine alone had no significant effect on RGC survival under hypoxic conditions (p=0.963).

## Discussion

We have demonstrated using an in vitro model of purified rat RGC culture that brimonidine is neuroprotective at the level of the RGC. The neuroprotective effect of brimonidine was present in three different stress situations—glutamate induced neurotoxicity, oxidative stress, and hypoxic stress. Brimonidine, at a 1 μM concentration, significantly increased RGC viability under all three stresses; these stresses have been implicated in the development of GON [2-5,8,14,16,17,35-37].

Baptiste et al. [15] used mixed retinal cell cultures of neurons and glia to demonstrate the neuroprotective effect of α2-adrenergic agonist UK14304 against glutamate-induced neurotoxicity. We believe our work is the first to report the neuroprotective effect of brimonidine on RGCs against glutamate-induced neurotoxicity, oxidative stress, and hypoxic stress, using purified rat RGC cultures. Our in vitro model of a purified rat RGC culture further adds to the evidence that brimonidine has neuroprotective effects not related to the lowering of IOP [4,14-17].

The neuroprotective pathways triggered by brimonidine were effectively blocked by the selective α2-adrenergic antagonist yohimbine. Previously, α2-adrenergic antagonists, like rauwolscine and yohimbine, were shown to reverse the neuroprotective effects of α2-adrenergic agonists in models of optic nerve injury and photoreceptor light-induced damage [17,38,39]. Mixed retinal cell culture experiments showed that brimonidine reduced glutamate-induced Ca^2+^ increases in retinal neurons in culture, the effect of which was reversed by yohimbine [40]. Our in vitro experiments with purified RGCs demonstrated that the neuroprotective effect of brimonidine is at the RGC level via α2-adrenergic receptors.

Our results showed a similar percentage protection effect over three different concentrations of brimonidine and under three different stresses. It is possible that a final common pathway of the three neurotoxic insults may be countered by the effect of brimonidine on α2-adrenergic receptors. The α2-adrenergic receptors are expressed in the inner plexiform and RGC layers of the retina in various mammalian species, such as rats and humans [41-45]. Activation of α2-adrenergic receptors may protect RGCs from experimental injury by preventing abnormal elevation of cytosolic free Ca^2+^ through modulation of the L-type Ca^2+^ channel or glutamate receptor activity [15,40,46]. In these studies, over 0.3 µM of brimonidine was needed to reduce cytosolic Ca^2+^ through the L-type Ca^2+^ channel, whereas over 3 µM of brimonidine was required for the modulation of glutamate-induced Ca^2+^ increase. Thus, the mechanism of neuroprotection observed in our study may be partly attributed to L-type Ca^2+^ channel modification because the effect was observed at a briminodine concentration of 1 µM.

Under our culture conditions, hypoxia mainly induced glutamate-independent apoptosis [19]. Anti-apoptotic pathways of α2-adrenergic receptor activation also include increased endogenous BDNF expression in RGCs, upregulation of basic fibroblast growth factor (bFGF), and induction of the anti-apoptotic genes *bcl-2 and bcl-xl* [47-49]. Thus, the neuroprotective effect of brimonidine on hypoxia-induced RGC death may be attributed to these mechanisms.

In contrast to glutamate- or hypoxia-induced neurotoxicity, oxidative stress induced by using B27 without antioxidative agents in the current model mainly induced necrosis by activation of the calpain/catepsin pathway [21]. The mechanism of brimonidine’s effect on the calpain/catepsin pathway deserves future study.

Pharmacologically, brimonidine can activate the α2-adrenergic receptor at a concentration of 2 nM or higher. Studies with monkeys showed that the vitreous humor brimonidine concentration was 82 nM after topical application of 0.2% brimonidine [50]. In humans, topically applied 0.2% brimonidine tartrate and 0.15% brimonidine purite twice or three times daily resulted in acquired vitreous levels of 185 nM and 19 nM brimonidine, respectively [51,52]. Thus, a topical or systemic application of brimonidine may be enough to activate α2-adrenergic receptors not only to reduce IOP but also to induce neuroprotective effects at the level of RGCs.

In summary, we first found that brimonidine acting via the α2-adrenergic receptor was neuroprotective on purified rat RGCs exposed to glutamate-induced neurotoxicity, oxidative stress, or hypoxic stress at concentrations of 10^−7^ M or higher. In an attempt to search for effective treatment of GON beyond IOP-lowering therapy, the potential of brimonidine or other α-2-selective adrenergic agonists to be able to affect not only the glutamate-induced apoptosis pathway but also the glutamate-independent apoptotic or calpain/catepsin-dependent necrotic pathway in RGCs may merit further study.
